# Immune activation is associated with decreased thymic function in asymptomatic, untreated HIV-infected individuals

**DOI:** 10.4102/sajhivmed.v17i1.445

**Published:** 2016-07-28

**Authors:** Thandiwe Manjati, Bongani Nkambule, Hayley Ipp

**Affiliations:** 1 Division of Haematology, Department of Pathology, Stellenbosch University, South Africa; 2 Division of Haematopathology, National Health Laboratory Service, Tygerberg Hospital, Cape Town, South Africa; 3 Department of Physiology, School of Laboratory and Medical Sciences, University of KwaZulu-Natal, South Africa

## Abstract

**Background:**

Reduced thymic function causes poor immunological reconstitution in human immunodeficiency virus (HIV)-positive patients on combined antiretroviral therapy (cART). The association between immune activation and thymic function in asymptomatic HIV-positive treatment-naive individuals has thus far not been investigated.

**Aims and objectives:**

To optimise a five-colour flow cytometric assay for measurement of thymic function by measuring recent thymic emigrants (RTEs) in treatment-naive HIV-infected patients and healthy controls and correlate results with levels of immune activation, CD4 counts and viral load.

**Methods:**

Blood obtained from 53 consenting HIV-positive individuals and 32 controls recruited from HIV prevention and testing clinic in Cape Town, South Africa. RTEs were measured (CD3^+^/CD4^+^/CD45RA^+^/CD31^+^/CD62L^+^) and levels were correlated with CD4 counts of HIV-infected individuals, log viral load and levels of immune activation (CD8^+^/CD38^+^ T-cells).

**Results:**

HIV-infected individuals had reduced frequencies of RTEs when compared to controls (*p* = 0.0035). Levels of immune activation were inversely correlated with thymic function (*p* = 0.0403), and the thymic function in HIV-infected individuals showed no significant correlation with CD4 counts (*p* = 0.31559) and viral load (*p* = 0.20628).

**Conclusions:**

There was impaired thymic function in HIV-infected individuals, which was associated with increased levels of immune activation. The thymic dysfunction was not associated with CD4 counts and viral load. Immune activation may result in inflammatory damage to the thymus and subsequent thymic dysfunction, and CD4 counts and viral load may not necessarily reflect thymic dysfunction in HIV.

## Introduction

### Background

Human immunodeficiency virus (HIV) infection is characterised by depletion of naive and memory CD4 T-cells due to its ability to damage both thymic and peripheral T-cell homeostasis. In addition, there is evidence of direct infection of thymocytes by HIV, which results in defective thymopoiesis and apoptosis of CD4 T-cells.^1,2^

Within a year of initiation of combined antiretroviral therapy (cART), the thymus of adult HIV-positive patients on cART expands,^3^ and evidence has shown that infected adults’ thymuses are still functional despite physiological involution. Therefore, the thymus plays a role in immune recovery or contributes to the lack of immune reconstitution in HIV-infected patients.^4^ In some studies, it has been shown that immune reconstitution in adults is mainly from the memory T-cell pool, whereas in children, it is mainly from the naive T-cell subset.^5^

### Literature review

Most studies that have assessed thymic output in HIV patients have shown reduced output prior to initiating cART and significant thymic output increase after therapy initiation.^4,5,6,7^

The ongoing viraemia prior to cART initiation may cause proliferation of recent thymic emigrants (RTEs) and result in differentiation to memory T-cells, which are more susceptible to HIV-1 infection than naive T-cells. This, in addition to reduced thymic function, reduces the number of RTEs in HIV infection.^8^ In addition to improved thymic function, another mechanism for the increase seen after initiation of cART is the release of lymphocytes that have been sequestered in lymphoid tissue during HIV replication.^7^ The effect of the increase due to memory T-cells redistributed from lymph nodes wanes after a few weeks of therapy, and the increase due to the production of new naive T-cells derived from thymopoiesis is maintained for a longer period.^6,9^

This entrapment of RTEs in lymphoid tissue was particularly seen in individuals with high viral loads.^10^

#### Immune activation in HIV infection

Chronic infections such as HIV infection are associated with chronic inflammation that may be systemic, affecting the whole body and leading to persistent immune activation with CD4 T-cell activation and turnover.^11,12^ Immune dysregulation causes a sustained increase in pro-inflammatory cytokines and erosion of immune defences. Persistent T-cell activation accelerates their maturation, cell growth and division. Immune activation in HIV leads to premature T-cell ‘burn out’ or clonal exhaustion and apoptosis.^11,12^

An important mechanism contributing to immune activation is the early damage to the gastrointestinal mucosa, which results in the ongoing translocation of microbes and microbial products into the systemic circulation.^12^ This is one of the causes of poor immune recovery after cART.^1,12,13^ Chronic immune activation in HIV results in poor immune recovery and thus poor outcome and faster disease progression as well as end organ complications.^12^ Chronic immune activation in HIV causes increased proliferation of thymocytes, which in the long term causes clonal exhaustion of T-cells and inflammatory damage to the lymphoid tissue.^1,12^ Patients with a large thymus have shown a better immune reconstitution when compared with those with a small thymus; therefore, thymopoiesis is important in immune reconstitution. Initiating therapy whilst thymic function is still good may be important in order to improve clinical outcomes.^13^ There are only few studies that have linked immune activation to thymic dysfunction in HIV-infected people.^14^

#### CD31 and CD62L cell markers

CD31 is a cell surface marker expressed preferentially by naive, T-cell receptor excision circle (TREC)-rich T-cells that have undergone a low number of T-cell divisions; therefore, CD31 can be used as a marker for RTEs.^15,16,17,18,19^ It is a 130-kDa transmembrane glycoprotein expressed by endothelial cells, platelets, monocytes, neutrophils and certain T-cell subsets. The average TREC content in CD3^+^/CD4^+^/CD45RA^+^/CD31^+^/CD62L^+^ T-cells is 18 times higher than in CD3^+^/CD4^+^/CD45RA^+^/CD31^-^/CD62L^+^ T-cells confirming the strong correlation between CD31-expressing naive CD4 T-cells and the presence of TREC.^15^ CD31 is downregulated on the majority of CD4 T-cells upon their transition to the memory phenotype.^15^ A progressive decrease of percentages and absolute numbers of RTEs has been found associated with ageing, and in addition, CD31 is downregulated during homeostatic expansion of naive T-cells.^13^

CD62L (L-selectin) is an adhesion molecule that allows T-cells to enter secondary lymphoid tissues via high endothelial venules. Early studies showed that bright CD62L expression was also a marker found on RTEs.^20,21,22^ It is expressed by naive T-cells and central memory T-cells and is absent on effector memory T-cells. It is upregulated by thymocytes^23^ and plays an essential role in lymphocyte homing to lymphoid tissue and sites of inflammation.^23,24^

Immune activation at the thymic site may result in inflammatory damage to the thymus and subsequent thymic dysfunction.^1^ This study investigated HIV-positive individuals who were not yet started on cART and measured thymic function using flow cytometry (CD31 and CD62L) as well as levels of immune activation (CD38 expression on CD8 T-cells).

## Research design

### Research approach and methods

This was a cross-sectional study of 53 consenting, untreated asymptomatic HIV-infected black South African adults and 32 uninfected controls aged > 21 years. The study was approved by the University of Stellenbosch, Faculty of Health Sciences, Human Research Ethics Committee (HREC N07/09/197). Patients and controls were recruited from an HIV prevention and testing clinic in Cape Town, South Africa. All individuals gave informed consent prior to their involvement in the study. Whole blood samples of 4 mL were collected from patients and controls into a heparin tube for measurement of RTEs and levels of immune activation, and 5 mL of blood was collected into an EDTA tube for CD4 count and viral load.

#### Measurement of recent thymic emigrant frequency using flow cytometry

The cell surface molecule expression was monitored by staining cells with the following fluorochrome-labelled monoclonal antibodies: CD31 FITC 5.6E, CD4 PE 13B8.2, CD45RA ECD J.33, CD62L PC5 DREG56 and CD3PC7 UCHT1. The optimal volumes of each antibody were mixed in a 5 µL cocktail and titrated in dilution experiments. The samples with 50 µL of heparinised blood and antibody cocktail were then incubated in the dark for 15 min at room temperature. Five hundred microlitres of phosphate-buffered saline (PBS) staining buffer was added and samples were analysed immediately. Data acquisition was done using the Beckman Coulter FC500 five-colour flow cytometer Calibur and CXP software.

#### Gating strategy

The cell type and size were identified by size, granularity and positive expression of surface markers CD31, CD62L and CD45RA specific for RTEs on CD4 T-cells. Initial gating was performed on CD3-positive population with low side scatter (T-cells). Secondary gating of CD45RA-positive cells and CD4-positive cells from the CD3-positive T-cells was done. CD3/CD45RA/CD4-positive naive T-cells were further analysed for CD31- and CD62L-positivity (RTEs). Controls or populations of cells negative for CD31 and CD62L were used to establish the ‘cut-off’ values for CD31- and CD62L-positivity. RTEs were described as CD3^+^/CD4^+^/CD45RA^+^/CD31^+^/CD62L^+^ T-cells. Levels of expression of these markers were correlated with CD4 counts, viral loads, levels of activation and patient’s age using Spearman’s R test.

#### CD4 T-cell count and viral load measurements

The BD MultiTEST CD3-FITC/CD8-PE/CD45-PerCP/CD4-APC reagent and BD TruCOUNT tubes (BD Biosciences, San Jose, CA) were utilised for the measurement of CD4 T-cell count. For viral load measurements, blood samples were collected into 5 mL EDTA tubes, which were centrifuged at 20°C at 300 g for 12 min. One millilitre of plasma was transferred into a Greiner Bio-one cryotube (Greiner Bio-One GmbH, Frickenhausen, Germany) and sent for viral load testing. The viral load assay performed was a NucliSensEasyQ^®^ HIV-1 v1.2 Viral Load Test (BioMerieux Inc., Boxtel, the Netherlands), which has a detection range of 1.60–6.7 log_10_ copies/mL. Both these tests were performed at the Division of Medical Virology, Faculty of Health Sciences, Stellenbosch University, which is accredited by the South African National Accreditation System (SANAS).

#### Measurements of the percentage of CD8+ T-cells expressing CD38

This measurement was performed using flow cytometry. Briefly, 50 µL of heparinised whole blood was stained with a titrated monoclonal antibody cocktail containing CD8 Per-CP, CD38 APC and CD3 FITC (BD Biosciences, San Jose, CA). Data acquisition was performed using a BD FACS Calibur instrument, and analysis was done using the BD Cell Quest Pro (Version 2) software.

#### Statistical analysis

The software used for statistical analysis was Graph Pad Prism version 5.0 for Windows, Graph Pad software, CA, USA. Mann–Whitney U test was used to determine % CD38^+^/8^+^ T-cells (levels of immune activation) and reported as the median and interquartile range, and unpaired *t*-test was used to determine % CD3^+^/CD4^+^/CD45RA^+^/CD62L^+^/CD31^+^ T-cells (RTEs) between the HIV-infected and control groups and reported as mean and standard deviation. Non-parametric data of HIV-positive individuals were analysed using Spearman’s *R* correlations. All *p*-values were considered as significant when < 0.05. These analyses were performed as single experiments.

## Results

The cohort consisted of 85 consenting individuals with similar ages and demographics. Of these, 53 were HIV-infected individuals and 32 uninfected controls with median ages (in years): control group 27 [23–34] versus HIV group 28 [25–35], *p* = 0.2113). Despite being clinically asymptomatic, the HIV-positive group had significantly lower CD4 counts when compared to the control group (*p* < 0.0001) as shown in Table 1.

**TABLE 1:** Participants’ demographics and disease parameters.

Parameters	HIV infected†		Uninfected controls‡		*p*-value
	
	*n*	Range	*n*	Range	
Median age (years)	28	25–35	27	23–34	0.2113
Male: female ratio	1:4	-	1:1	-	-
Median CD4 (cells/mm^3^)	354.0	208–503	828.0	644.8–1123	<0.0001
Log viral load	4.2705	-	-	-	-
CD38^+^/8^+^ (% cells gated)	26.14	6.800–12.64	8.610	17.62–40.38	<0.0001

† *n* = 53;

‡ *n* = 32.

**FIGURE 1:**
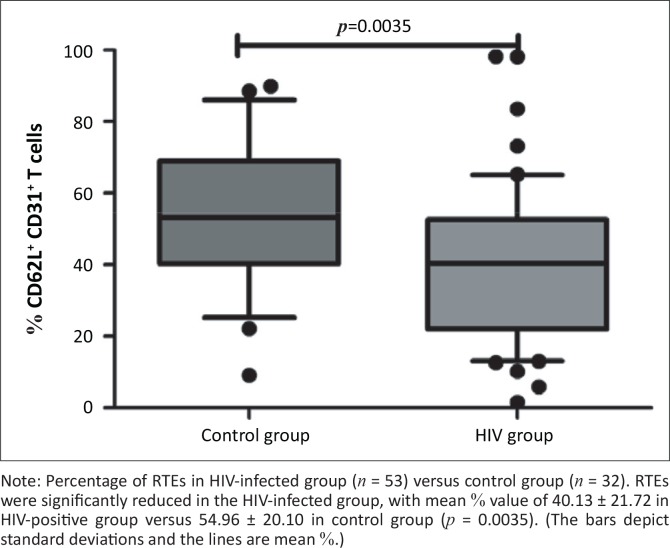
Baseline levels of per cent of CD4+/CD45RA+/CD62L+/CD31+ T-cells in HIV-positive versus control groups.

**FIGURE 2:**
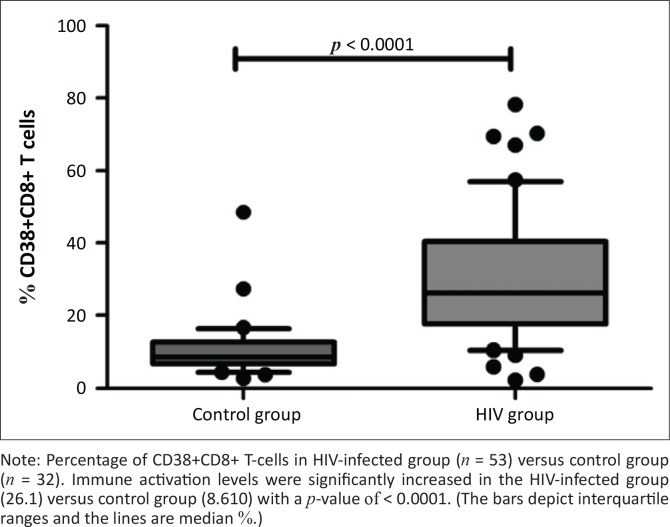
Baseline levels of % of CD38+/CD8+ T-cells in HIV-positive versus control groups.

RTEs were significantly reduced in the HIV-infected group, with mean % value of 40.13 ± 21.72 in HIV-positive group versus 54.96 ± 20.10 in control group (*p* = 0.0035) (as shown in Figure 1). Immune activation levels were significantly increased in the HIV group as determined by the marker of activation, CD38^+^/8^+^-positive T-cells, which was increased in the HIV-infected group (26.1) versus control group (8.610) with a *p*-value of < 0.0001 (as shown in Figure 2).

Further analysis of HIV group was done using Spearman’s R correlations for non-parametric data (as shown in Table 2).

**TABLE 2:** Spearman’s R correlations for HIV group.

Non-parametric data for HIV group	Non-parametric data for HIV group	*R*	*p*-value
CD4 count	RTEs	0.14335	0.31559
CD4 count	Log viral load	-0.2970	0.0425*
CD4 count	Age	-0.2371	0.0938
CD4 count	Levels of immune activation	-0.3977	0.0035*
RTEs	Levels of immune activation	-0.2910	0.0403*
RTEs	Age	-0.1494	0.3054
Levels of immune activation	Log viral load	0.4311	0.0027*
Levels of immune activation	Age	-0.1642	0.2543
Log viral load	Age	-0.0299	0.8449
RTEs	Log viral load	-0.19204	0.20628

* *p*-values are significant: < 0.005.

Log viral load correlated inversely with the CD4 counts, whereas there was a direct correlation between log viral load and levels of immune activation (*r* = 0.431, *p* = 0.0027). The CD4 count correlated inversely with levels of immune activation (*r* = -0.397, *p* = 0.0035).

Levels of immune activation correlated inversely with the number of RTEs (*p* = 0.0403) in this HIV-positive group. There were no significant correlations between age in the HIV-positive cohort and other parameters such as CD4 counts, viral load, levels of immune activation and RTEs in this group, and RTEs did not show a significant correlation with the CD4 counts or the log viral load.

## Discussion

RTEs refer to T-cells that have undergone only a few cellular divisions after leaving the thymus.^25,26^ These naive T-cells have recently exited the thymus and have not undergone peripheral T-cell proliferation and antigen selection. They have high TRECs and their numbers in peripheral blood depend on the magnitude of thymic export.^17^ Therefore, measuring the number of RTEs allows assessment of the thymic contribution to the peripheral T-cell pool. TREC concentrations in RTEs are affected by factors such as lymphopenic conditions, where absolute numbers of TRECs can be influenced by dilution factors due to peripheral expansion of naive and memory T-cells, for example, after haematopoietic stem cell transplantation or in HIV-infected patients after cART^15^; ageing also decreases the number of TRECs 50–100 fold.^15^ However, controversy exists as to whether TREC concentrations are a good marker for RTE, because TREC concentrations are also affected by peripheral T-cell turnover events, such as T-cell division and death.^17,27,28^ In HIV infection and other lymphopenic diseases, T-cell homeostasis is maintained by the ability of the thymus to export new naive T-cells.^29^ Therefore, thymic function can be monitored in conditions that influence T-cell depletion and reconstitution, such as HIV-1 infection, bone marrow transplantation and immunosuppressive therapy.^30^

In this study, we measured RTEs using flow cytometry, with particular use of the marker CD31. We designed and optimised a flow cytometry panel that could be used as a measure of thymic function in asymptomatic, treatment-naive HIV-infected patients. We showed that RTEs were significantly decreased in this group, even with CD4 counts median of 354 cells/mm^3^ (which is > 350 cells/mm^3^). The treatment cut-off value in South Africa had been below 350 cells/mm^3^ until recently. Furthermore, we showed for the first time to our knowledge, levels of RTEs by flow cytometry correlated inversely with immune activation levels in untreated HIV-infected individuals. This supports the concept that ongoing immune activation has a damaging effect on thymic function in untreated HIV infection. Interestingly, RTE levels did not correlate with viral load nor CD4 count suggesting that these parameters do not necessarily reflect thymic dysfunction at this stage of the disease.

Most studies that have assessed thymic output in HIV patients have shown reduced output prior to initiating cART and significant thymic output increase after therapy initiation.^4,6,7^ However, these studies have not shown the difference between HIV-positive individuals and uninfected controls, and CD4 counts were lower (200–300 cells/mm^3^) and viral log levels were higher (4.8–5.0 log copies/mL) in comparison with our HIV-positive group. In addition, the studies that compared untreated HIV-positive individuals with uninfected controls were conducted in children.

The results of our study have highlighted previous literature findings, wherein untreated HIV-positive individuals have increased immune activation when compared to uninfected controls.^11,31^ Furthermore, we showed that the higher levels of immune activation (as demonstrated by the increased percentage of CD38+/CD8+ T-cells) were significantly associated with lower CD4 counts and higher viral loads.

In untreated HIV-positive individuals, the activation marker CD38 indicates rapid clinical progression of disease and death more strongly than CD4+ T-cell counts and plasma HIV RNA levels.^32^ There is evidence that thymic output is required to maintain efficient gut mucosal defence.^32^ Bourgeois et al. showed in a mouse model of chemical thymomectomy that loss of gut immunity in HIV infection, particularly Th17 cells, leads to loss of barrier integrity and subsequent bacterial translocation and microbial products into circulation, which results in chronic immune activation.^29^ These findings suggest that augmenting thymic output can be used to correct aberrant activation caused by HIV infection or HIV-induced microbial translocation.^29,32^

Our study findings highlight the concept that ongoing immune activation impacts thymic production of naive T-cells thus resulting in depletion of the naive T-cell pool seen in HIV infection. This is supported by a study conducted by Bandera et al. of ex-vivo thymuses from HIV-positive individuals and uninfected controls.^1^ They analysed markers of T-cell differentiation and activation at different stages of thymopoiesis and found a significantly higher proportion of activated and proliferating thymocytes at all stages of thymopoiesis in HIV-infected patients compared to controls. They suggested that this increased activation and proliferation of thymocytes at the thymic site might in the long term cause clonal exhaustion of T-cells (‘burn out’) and damage to lymphoid tissue.^1^ The immune activation at the thymic site is likely caused by bystander mechanisms and sustained by homeostatic proliferation and may result in inflammatory damage leading to thymic dysfunction.^1^ Some studies have reported that HIV can directly infect the thymus thereby compromising its integrity.^2,33^ Reduced T-cell restoration has been reported to be caused by the effects of atrophy or shrinkage of the thymus. Using the simian immunodeficiency virus-infected rhesus macaques, Wykrzykowska et al. showed that in the first 7 days of infection, the thymus has a regenerative capacity with increased cell proliferation; however, from 24 weeks after infection there was evidence of severe thymic damage.^2^ This group concluded that the regenerative capacity of the thymus is limited in HIV infection. Early thymic dysfunction in children is also related to rapid progression to acquired immune deficiency syndrome (AIDS).^34^ Middle-aged people with HIV showed evidence of immunosenescence (ageing of the immune system) resembling that of HIV-negative individuals two decades older. The ongoing immune activation and inflammation due to constant stimulation by HIV or other chronic infections accelerates the process of immunosenescence.^11,35^

Our findings showed an association between thymic function in HIV infection and levels of immune activation. There seems to be a vicious cycle that results from immune activation causing damage to the thymus and thus causing dysfunctional thymic output. In turn, the reduced thymic output results in immune activation due to loss of mucosal barrier integrity and subsequent bacterial translocation and microbial products into circulation.

## Conclusion

There is mounting evidence of the contribution by the adult thymus to immune reconstitution in HIV infection.^1,35^ Larger thymic size was associated with higher CD4 counts and higher thymic outputs.^35^ We therefore suggest that initiating cART in patients with poor thymic function could be associated with poor immunological response. This study supports earlier initiation of cART, before thymic damage or irreversible dysfunction sets in.

This use of flow cytometry markers of RTEs in conjunction with a marker of immune activation would be a valuable addition to the assessment of thymic function in these individuals. This panel could then be used to follow up thymic output after initiation of therapy. Other methods used to estimate thymic function include measurements of thymic volume using computed tomography (CT) scan and evaluation of TREC-bearing cells by quantitative polymerase chain reaction (PCR).^3^ However, these techniques are expensive and TREC data may be difficult to interpret.^36^ This study presents a robust alternative method that can be used simultaneously to measure the levels of immune activation.

Further studies on the measurement of thymic function and levels of immune activation in HIV-positive patients with CD4 counts > 500 cells/mm^3^ are recommended. This would further establish whether immune dysfunction due to decreased thymic output is occurring at even earlier stages of the infection. These studies would support the earlier intervention with ART for the protection of thymic function and better immunologic recovery. It will be important to develop novel therapeutic strategies to limit immune activation and in doing so, protect the thymus from inevitable damage and dysfunction.

## Potential study limitations

This study was conducted on the black South African population group and therefore may not be representative of other racial groups in South Africa. The HIV cohort consisted of more females versus males. It is not known whether the hormonal differences between the two sexes can influence thymic function. It was also unknown how long each HIV-positive patient had been infected without treatment, and therefore, we do not know if the length of infection influences thymic output. The HIV-infected individuals are defined as being asymptomatic. Symptoms were determined by clinical questioning and examination but no further special investigations such as CXR were performed; so subclinical infections may have been missed. Finally, this was a small study group and a follow-up study on a larger cohort is considered.
